# Long non-coding RNA FAM99A modulated YAP1 to affect trophoblast cell behaviors in preeclampsia by sponging miR-134-5p

**DOI:** 10.1590/1414-431X20209732

**Published:** 2020-10-21

**Authors:** Bingnv Xu, Xiaofang Geng, Xiaodan Liu, Ying Liu

**Affiliations:** Department of Obstetrics, Maternal and Child Health Hospital Dongchangfu District, Liaocheng, Shandong, China

**Keywords:** lncRNA FAM99A, miR-134-5p, YAP1, Preeclampsia, Apoptosis

## Abstract

Preeclampsia (PE) is a complex pregnancy syndrome. Convincing evidence indicates that long non-coding RNAs (lncRNAs) are involved in the pathogenesis of PE. This research mainly investigated the mechanism of family with sequence similarity 99 member A (FAM99A) in PE. The expressions of FAM99A, miR-134-5p, and YAP1 were detected by quantitative real-time polymerase chain reaction (qRT-PCR). Cell apoptosis, migration, and invasion were detected by flow cytometry or transwell assay. The interaction between miR-134-5p and FAM99A or YAP1 was confirmed by dual-luciferase reporter assay. The protein expression of YAP1 was determined by western blot assay. FAM99A and YAP1 were significantly up-regulated, and miR-134-5p was significantly down-regulated in PE tissues (n=30). miR-134-5p was verified as a candidate of FAM99A and YAP1. FAM99A promoted cell metastasis, but reduced apoptosis in HTR8/SVneo cells by regulating miR-134-5p. miR-134-5p down-regulated YAP1 expression to suppress cell metastasis, while it induced apoptosis in HTR8/SVneo cells. FAM99A positively modulated YAP1 expression by sponging miR-134-5p. FAM99A modulated YAP1 to accelerate cell migration and invasion, and inhibited cell apoptosis in PE cells by sponging miR-134-5p. The novel regulatory network may shed light on the pathogenesis of PE.

## Introduction

Preeclampsia (PE) is a complicated pregnancy disorder diagnosed by high blood pressure and protein in urine accompanying high maternal morbidity and mortality of (1–3). In healthy pregnant women, extravillous trophoblasts (EVTs) invade from the decidua and myometrium and reach the spiral arteries to provide nutrients to the fetus (4). In PE patients, EVT invasion to the myometrium is impaired (5). However, the mechanism of this phenomenon has been rarely documented. Long non-coding RNAs (lncRNAs) are a family of long RNAs (>200 nt) without translation capacity that affect target gene expression at the post-transcription stage (6). In PE, many lncRNAs have been reported to be dysregulated and affect cell behaviors of trophoblasts. For example, Chen et al. (7) demonstrated that lncRNA MALAT-1 was lowly expressed in PE, and MALAT-1 depletion blocked cell growth but induced cell apoptosis *in vitro*. Similar results were also reported for MEG3 (8), TUG1 (9), and PVT1 (10). The family with sequence similarity 99 member A (FAM99A) gene is located on chromosome 11 and is associated with maternal circulating lipid profile in pregnant women (11). A previous study revealed that the low expression of FAM99A in PE inhibited cell growth and metastasis and accelerated cell apoptosis *in vitro* (12). However, the potential mechanism of FAM99A is still undefined in PE.

MicroRNAs (miRNAs), a class of small RNAs (∼22 nt) with no translation ability, could down-regulate target genes that are expressed at messenger RNA (mRNA) stage (13). Convincing evidence shows that many miRNAs are aberrantly expressed in PE. For instance, miR-210 overexpression inhibited cell metastasis in PE (14). Other miRNAs, like miR-17, miR-20a, and miR-20b, are enhanced in PE (15). Moreover, miR-134 is significantly up-regulated in the first trimester of PE plasma exosomes, and miR-134 retarded cell metastasis by targeting ITGB1 in PE (16). Yes-associated protein 1 (YAP1) is implicated in Hippo signaling pathway, which is associated with tissue homeostasis and tumor formation (17). For instance, lncRNA PFAR could enhance lung fibrosis by regulating miR-138/YAP1 axis (18). Moreover, a recent study indicated that YAP1 regulates LINC00152 to inhibit the progression of colorectal cancer (19). Sun et al. (20) reported that YAP1 is elevated in PE and facilitates cell metastasis, but impedes apoptosis. However, the underlying mechanism of miR-134-54p and YAP1 is rarely reported in PE. In this research, the mechanism of FAM99A was mainly explored. The underlying regulatory network may provide a new perspective in PE development.

## Material and Methods

### Tissue collection

This research was approved by the Ethics Committee of Maternal and Child Health Hospital of Dongchangfu District of Liaocheng (China) and was carried out according to the Declaration of Helsinki Principles. Thirty PE placenta tissue samples and twenty normal pregnant placenta tissue samples were obtained from the above hospital. All samples were frozen at -80°C until further use. Written informed consents were provided from all participants. The clinical characteristics of PE and normal women are listed in Table 1.

**Table 1:** Clinical characteristics of preeclampsia (PE) patients and normal pregnant women.

Parameters	Control (n=20)	PE (n=30)	P value
Maternal age at delivery, years	27.42±1.23	28.55±0.95	0.352
Gestational age, weeks	37.68±4.20	35.25±5.36	<0.01
Proteinuria, g/24 h	-	3.95±1.18	NA
Systolic blood pressure, mmHg	113.52±2.16	167.52±2.69	<0.01
Diastolic blood pressure, mmHg	73.52±3.20	104.32±4.24	<0.01
Fetal birth weight, g	3415.1±352.06	2541.32±641.35	<0.01

Data are reported as means±SD (Student's *t*-test).

### Cell culture and transfection

Human placenta trophoblast cell line HTR8/SVneo was bought from Procell (China) and cultivated in RPMI-1640 medium (PM150110A; Procell) with 5% fetal bovine serum (FBS; Procell) in 5% CO_2_ in an incubator at 37°C.

Small interfering RNA targeting FAM99A (si-FAM99A#1, si-FAM99A#2, and si-FAM99A#3), FAM99A overexpression plasmid (FAM99A), YAP1 overexpression plasmid (YAP1), miR-134-5p mimics (miR-134-5p), miR-134-5p inhibitor (anti-miR-134-5p), and their corresponding negative controls (si-NC, miR-NC, and anti-miR-NC) were all obtained from Sangon Biotech (China). Cell transfection was conducted using Lipofectamine 2000 (Invitrogen, USA).

### Quantitative real-time polymerase chain reaction (qRT-PCR)

The extracted RNA samples were synthesized to cDNA with random primers using an RT kit (TaKaRa, China). The quantitative PCR was carried out using a SYBR kit (TaKaRa). The levels of FAM99A, YAP1, and miR-134-5p were normalized by GAPDH or U6, and analyzed with the 2^-ΔΔCt^ method. All primers were bought from RiBoBio (China) and are as follows: FAM99A: (F, 5′-GGCCCACGACATCAGGTAAA-3′, R, 5′-TACGAAATGTCTCGGCGGTC-3′); miR-134-5p: (F, 5′-ACACTGCATCCTGGCAATTC-3′, R, 5′- CGTGGTGAATCGAGACTCAC-3′); YAP1: (F, 5′-CAGGAATTATTTCGGCAGGA-3′, R, 5′-CATCCTGCTCCAGTGTAGGC-3′); GAPDH: (F, 5′-TGGAAGGACTCATGACCACA-3′, R, 5′-TTCAGCTCAGGGATGACCTT-3′); and U6: (F, 5′-CTCGCTTCGGCAGCACA-3′, R, 5′-AACGCTTCACGAATTTGCGT-3′).

### Flow cytometry analysis of cell apoptosis

An apoptosis detection kit (Fcmacs, China) was used to evaluate the apoptotic rate. The lysate cell samples were incubated at 4°C to label with Annexin V-fluorescein isothiocyanate (FITC) and propidium iodide (PI) for 10 min in the dark. The apoptotic rate was assessed by flow cytometry (FACScan; BD Biosciences, USA).

### Transwell assay

Transwell chambers (Corning, USA) were used to detect the migrated and invaded capacities of transfected HTR8/SVneo cells. For cell migration, RPMI-1640 medium with 5% FBS was added to the lower chamber, while the transfected HTR8/SVneo cells were injected into the upper chamber with RPMI-1640 medium with no-serum. Following 24-h cultivation, the migrated cells in the lower chamber were fixed with methanol, stained with 0.1% crystal violet, and then counted in 10 random fields using a light microscope. For cell invasion, the upper chamber was pre-coated with a Matrigel matrix (Corning).

### Dual-luciferase reporter assay

The amplified wild type and mutant fragments of FAM99A and YAP1 3′UTR were inserted into the pGL3 vector (Promega, USA) to construct luciferase reporter, namely FAM99A-WT, FAM99A-MUT, YAP1-WT, or YAP1-MUT. The co-transfection of luciferase reporter and miRNA was performed as described above. The luciferase activity was tested using Dual-Lucy Assay kit (Solarbio, China).

### Western blot assay

The extracted protein samples from HTR8/SVneo cells were separated by sodium dodecyl sulfonate-polyacrylamide gel electrophoresis (SDS-PAGE) and then transferred onto a polyvinylidene fluoride (PVDF) membrane. The membrane was blocked in skim milk for 2 h and incubated with anti-YAP1 primary antibody (Bioss, China) at 4°C overnight. Following 2-h incubation with secondary antibody, the chemiluminescence was examined using an ECL kit (Beyotime, China).

### Statistical analysis

All data analyses were performed using GraphPad Prism 7 (GraphPad Inc., USA). Quantitative data were repeated at least three times and reported as means±SD. The difference between two groups was analyzed by Student's *t*-test, while the difference among multiple groups was compared by one-way analysis of variance (ANOVA). Statistical significance was considered as P<0.05.

## Results

### FAM99A and miR-134-5p expression in PE tissues

**Figure 1:**
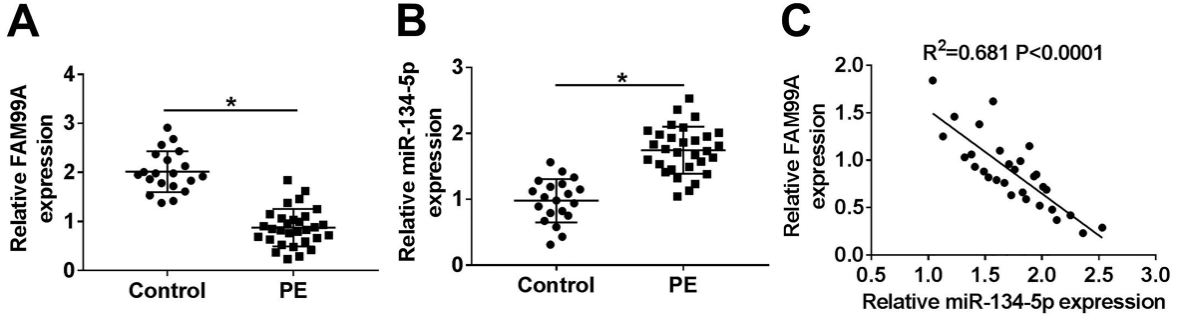
FAM99A was down-regulated, but miR-134-5p was significantly up-regulated in preeclampsia (PE) tissues. **A** and **B**, The levels of FAM99A (**A**) and miR-134-5p (**B**) in PE tissues and normal pregnant placenta tissues were measured by qRT-PCR. **C**, A correlation between FAM99A and miR-134-5p was detected. *P<0.05 (Student's *t*-test).

As presented in Figure 1A and B, the relative expression of FAM99A was increased, while miR-134-5p was decreased in PE tissues compared to that in the normal group. The scatter chart shows that FAM99A was negatively correlated with miR-134-5p (Figure 1C). In addition, PE patients were associated with some clinical characteristics, including shorter gestational age, higher systolic and diastolic blood pressure, and lower fetal birth weight (Table 1).

### FAM99A affected cell metastasis and apoptosis in HTR8/SVneo cells

**Figure 2:**
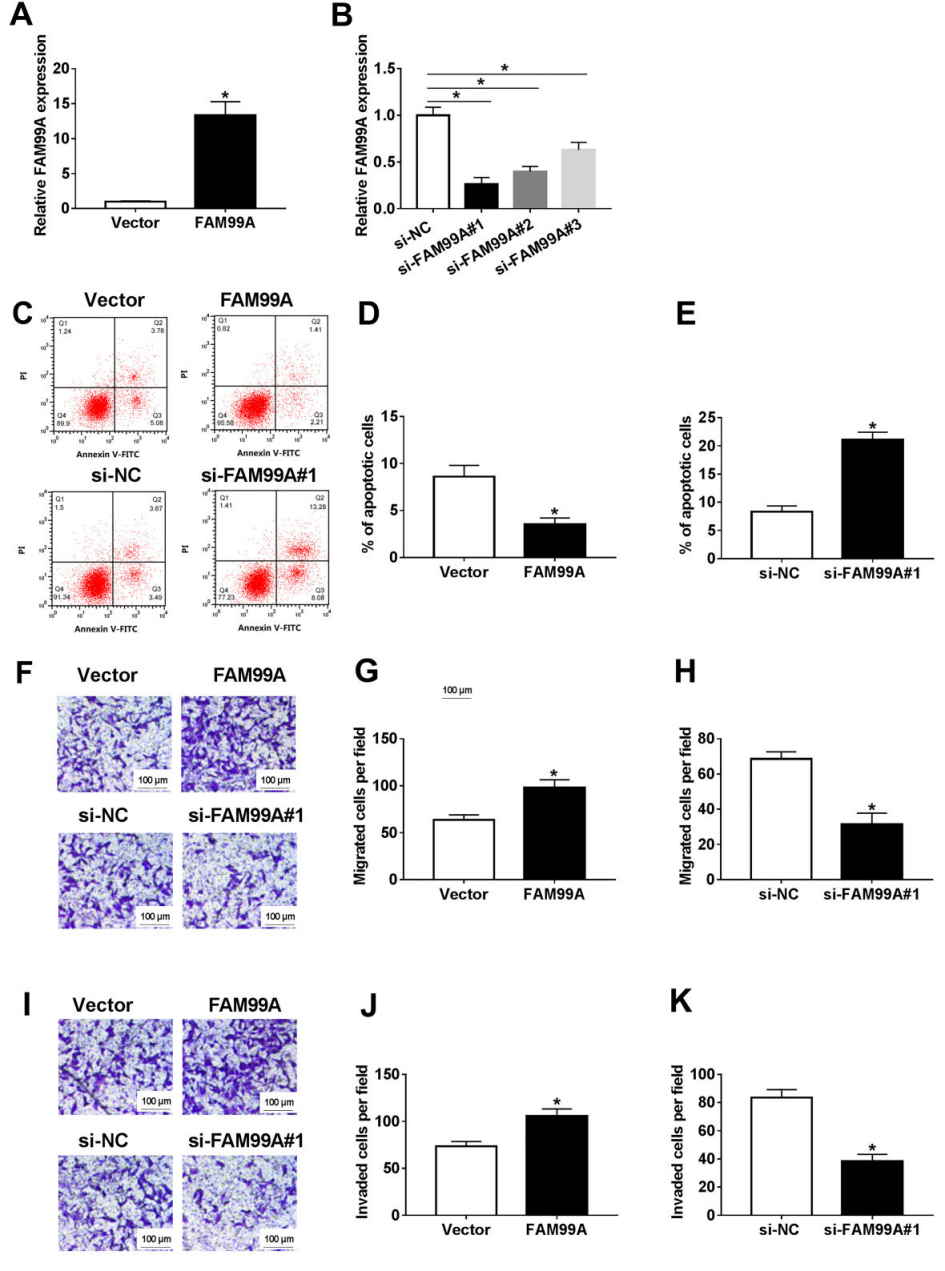
**A**, The level of FAM99A in HTR8/SVneo cells transfected with FAM99A mimics or negative control was detected by qRT-PCR. **B**, The level of FAM99A in HTR8/SVneo cells transfected with si-FAM99A (si-FAM99A#1, si-FAM99A#2, and si-FAM99A#3) or negative control si-NC was detected by qRT-PCR. **C-K**, The HTR8/SVneo cells were transfected with FAM99A mimics, si-FAM99A, or negative controls (Vector, si-NC). **C-E**, The apoptotic rate was evaluated by flow cytometry. **F-K**, The migrated and invaded cells per field were calculated via Transwell assay (scale bar 100 μm). Data are reported as means±SD. *P<0.05 (Student's *t*-test or ANOVA).

The level of FAM99A was elevated in HTR8/SVneo cells transfected with FAM99A mimics, while FAM99A was reduced in HTR8/SVneo cells transfected with si-FAM99A (Figure 2A and B). Furthermore, flow cytometry results showed that the apoptotic rate was significantly decreased by FAM99A overexpression in HTR8/SVneo cells, whereas significantly accelerated in si-FAM99A group (Figure 2C-E). Furthermore, the migrated and invaded cells were significantly boosted in FAM99A group, but significantly decreased in si-FAM99A group (Figure 2F-K). These data revealed that FAM99A promoted cell migration and invasion but impeded cell apoptosis in HTR8/SVneo cells, while FAM99A silencing exhibited the opposite results.

### MiR-134-5p was a direct target of FAM99A

**Figure 3:**
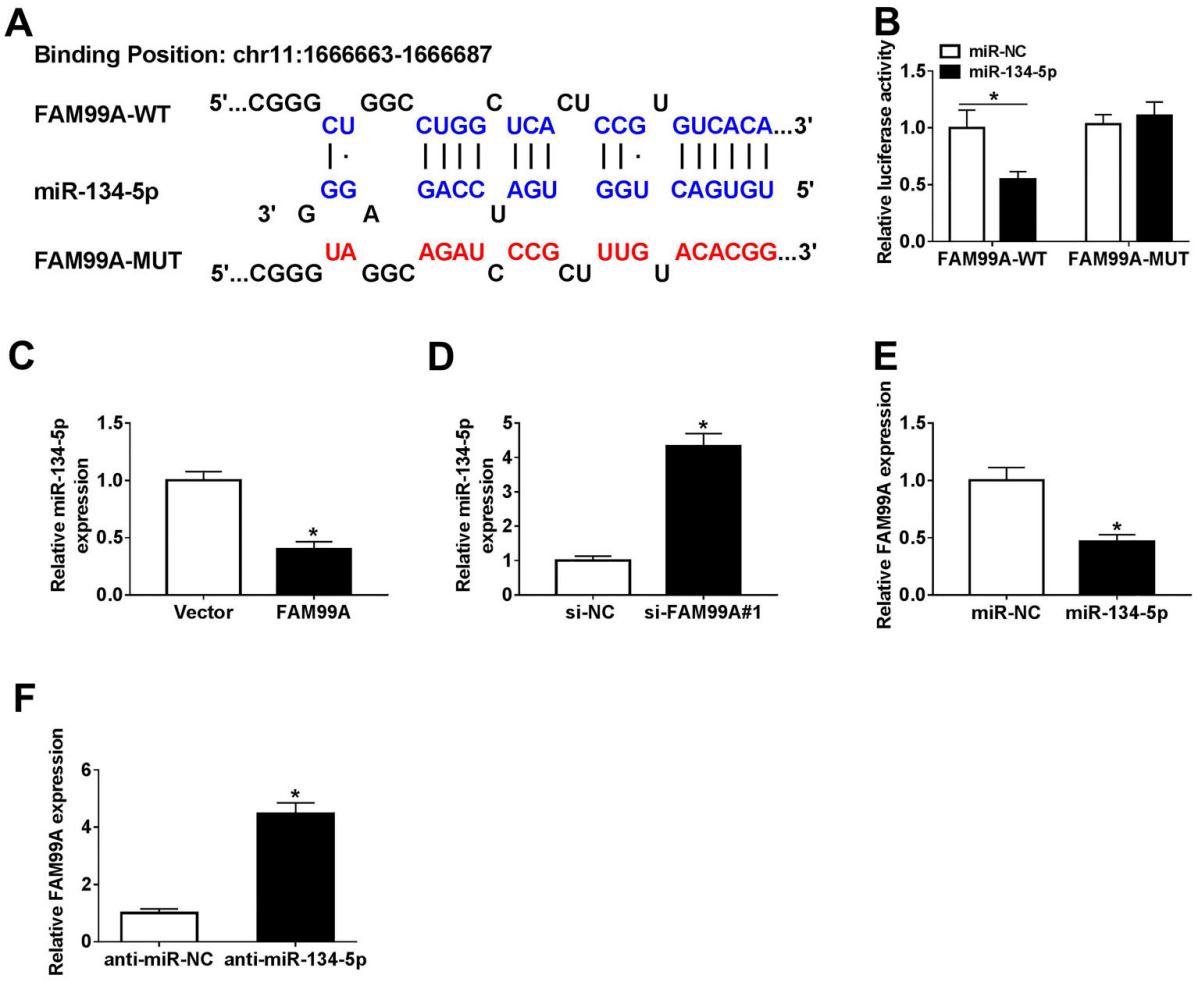
**A**, The complementary binding sites between FAM99A wild type (WT) and miR-134-5p are shown, as well as the sequences of FAM99A mutant (MUT). **B**, The luciferase activity of FAM99A-WT or FAM99A-MUT in HTR8/SVneo cells transfected with miR-134-5p or miR-NC was assessed via dual-luciferase reporter assay. **C** and **D,** The level of miR-134-5p in HTR8/SVneo cells transfected with FAM99A, si-FAM99A#1, or negative controls was measured by qRT-PCR. **E** and **F**, The level of FAM99A in HTR8/SVneo cells transfected with miR-134-5p, anti-miR-134-5p, or negative controls (NC) was detected via qRT-PCR. Data are reported as means±SD. *P<0.05 (Student's *t*-test).

To explore the underlying mechanism of FAM99A in PE, DIANA Tools (http://carolina.imis.athena-innovation.gr/diana_tools) was used to predict the putative target of FAM99A. The results showed that miR-134-5p had the complementary sequences with FAM99A (Figure 3A). Furthermore, the luciferase activity of FAM99A-WT reporter was significantly reduced in HTR8/SVneo cells transfected with miR-134-5p compared to that the in miR-NC (negative control) group, while the luciferase activity of FAM99A-MUT had no obvious change (Figure 3B). Moreover, the level of miR-134-5p was down-regulated in HTR8/SVneo cells transfected with FAM99A, but up-regulated in the si-FAM99A group (Figure 3C and D). In addition, the transfection of miR-134-5p resulted in a decrease of FAM99A expression, while the introduction of anti-miR-134-5p contributed to an increase of FAM99A level (Figure 3E and F). Taken together, miR-134-5p negatively interacted with FAM99A.

### Effect of FAM99A on cell metastasis and apoptosis

**Figure 4:**
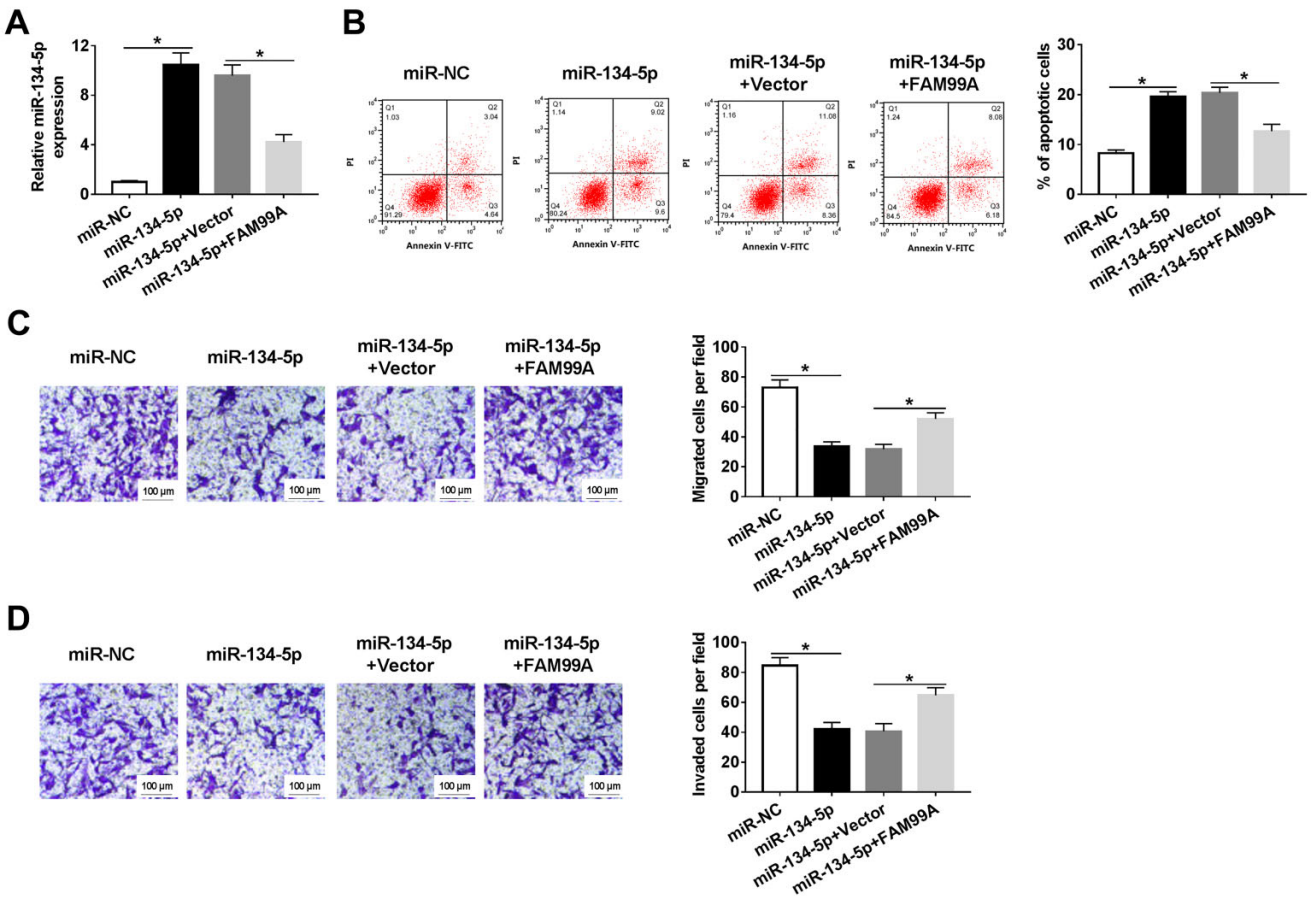
HTR8/SVneo cells were transfected with miR-NC (negative control), miR-134-5p, miR-134-5p+Vector, or miR-134-5p+FAM99A. **A**, The level of miR-134-5p was detected by qRT-PCR. **B**, The apoptotic rate was assessed by flow cytometry. **C** and **D**, The migrated and invaded cells per field were calculated by Transwell assay (scale bar 100 μm). Data are reported as means±SD. *P<0.05 (ANOVA).

As shown in Figure 4A, FAM99A overexpression partly inhibited the level of miR-134-5p in HTR8/SVneo cells transfected with miR-134-5p. Furthermore, the apoptotic rate was increased in the miR-134-5p group, which was blocked by FAM99A overexpression (Figure 4B). Transwell assay demonstrated that FAM99A overexpression counteracted the suppressive effects of miR-134-5p mimic on cell migration and invasion (Figure 4C and D). These data suggested that FAM99A could reverse the inhibition effect of miR-134-5p on cell migration, invasion, and the promotion effect on cell apoptosis in HTR8/SVneo cells.

### YAP1 negatively interacted with miR-134-5p

**Figure 5:**
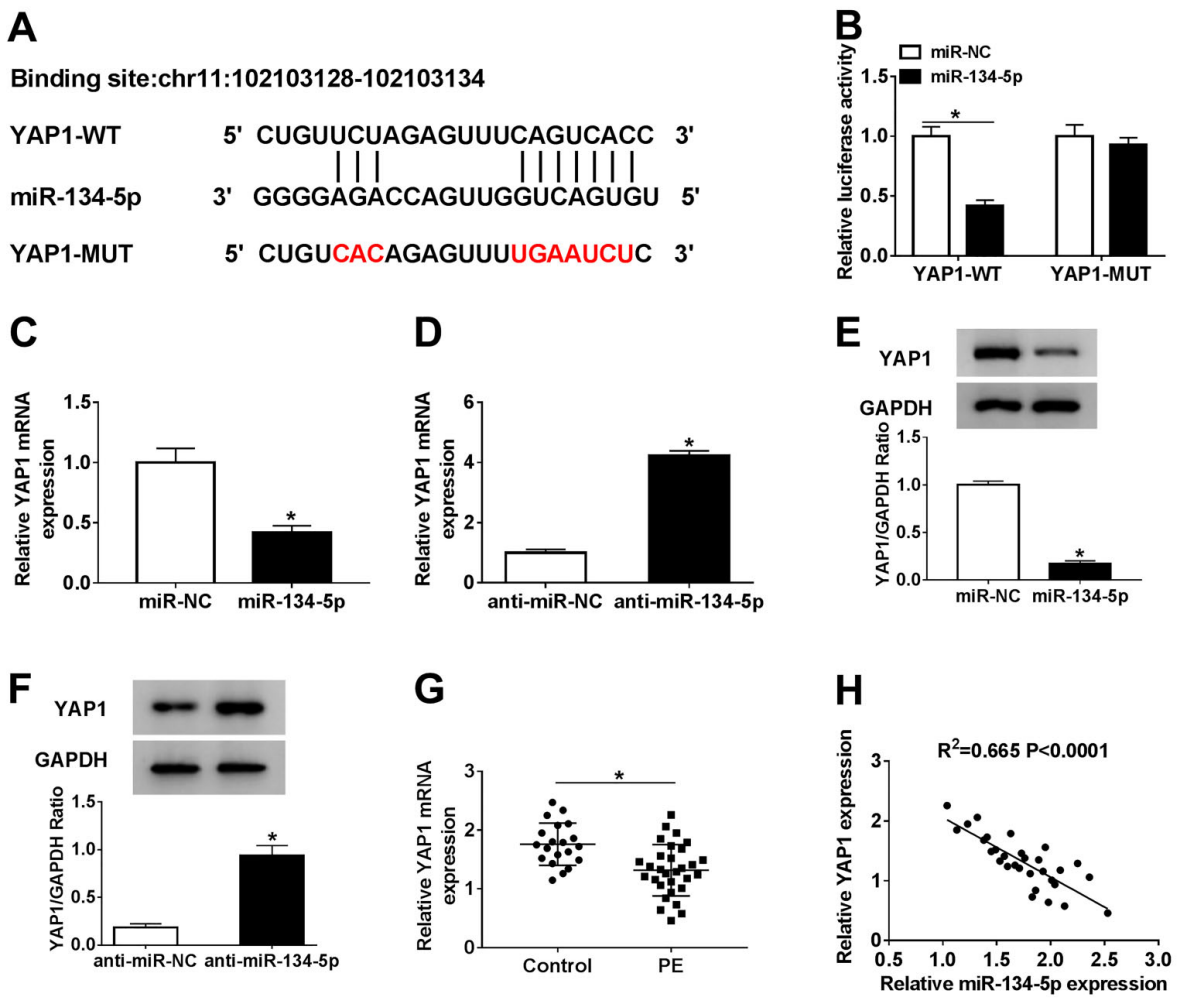
A, The complementary binding sites between YAP1 wild type (WT) and miR-134-5p are shown, as well as the sequences of YAP1 mutant (MUT). **B**, The luciferase activity of YAP1-WT or YAP1-MUT in HTR8/SVneo cells transfected with miR-134-5p or miR-NC (negative control) was assessed via dual-luciferase reporter assay. **C** and **D**, The level of YAP1 in HTR8/SVneo cells transfected with miR-134-5p, anti-miR-134-5p, or NC was measured by qRT-PCR. **E** and **F**, The protein level of YAP1 in HTR8/SVneo cells transfected with miR-134-5p, anti-miR-134-5p, or NC was detected via western blot. **G**, The level of YAP1 in preeclampsia (PE) tissues and normal pregnant placenta tissues was tested by qRT-PCR. **H**, The correlation between YAP1 and miR-134-5p was measured. Data are reported as means±SD. *P<0.05 (Student's *t*-test).

To investigate the molecular mechanism of miR-134-5p in PE, starBase online database (http://starbase.sysu.edu.cn/starbase2/) was used to search for potential targets of miR-134-5p. The results indicated that there were complementary binding sites between YAP1 3′UTR and miR-134-5p (Figure 5A). Moreover, miR-134-5p significantly decreased the luciferase activity of YAP1-WT, but the luciferase activity of YAP1-MUT reporter had no evident change (Figure 5B). Meanwhile, the mRNA and protein levels of YAP1 were both reduced in HTR8/SVneo cells transfected with miR-134-5p, but were increased in the anti-miR-134-5p group (Figure 5C-F). Furthermore, the level of YAP1 was markedly down-regulated in PE tissues and negatively correlated with miR-134-5p level (Figure 5G and H). These results indicated that YAP1 was a direct target of miR-134-5p.

### YAP1 effect on cell migration, invasion, and apoptosis in HTR8/SVneo cells regulated by miR-134-5p

**Figure 6:**
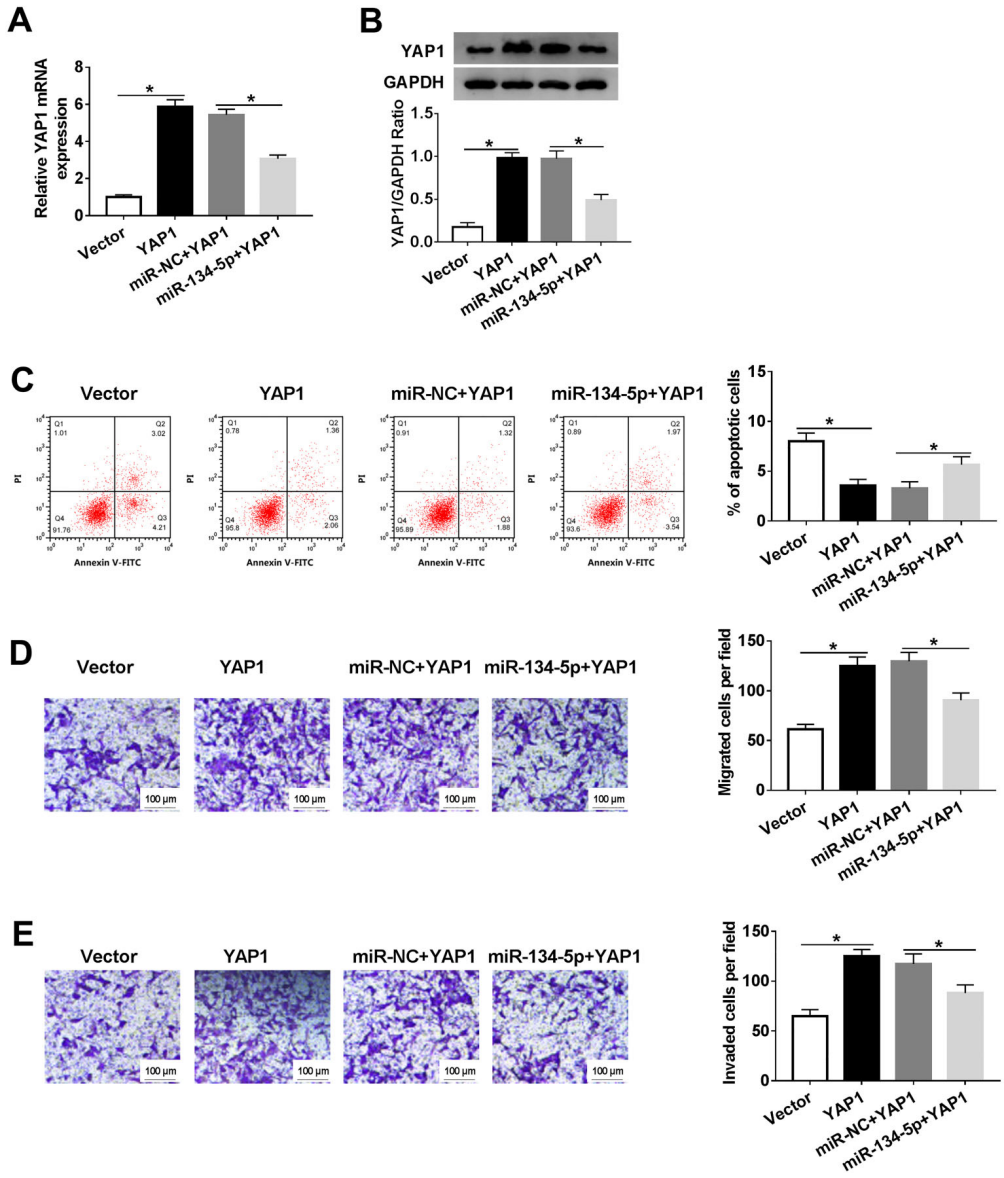
A-**E**, HTR8/SVneo cells were transfected with Vector, YAP1, miR-NC (negative control)+YAP1, or miR-134-5p+YAP1. **A**, The level of YAP1 was detected by qRT-PCR. **B**, The protein level of YAP1 was examined via western blot. **C**, The apoptotic rate was assessed through flow cytometry. **D** and **E**, The migrated and invaded cells per field were calculated by Transwell assay (scale bar 100 μm). Data are reported as means±SD. *P<0.05 (ANOVA).

To further explore the functions of YAP1 and miR-134-5p, HTR8/SVneo cells were co-transfected with miR-134-5p and YAP1. The level of YAP1 was enhanced in HTR8/SVneo cells transfected with YAP1, and weakened in HTR8/SVneo cells co-transfected miR-134-5p and YAP1 (Figure 6A). Western blot assay showed similar results (Figure 6B). Furthermore, the apoptotic rate impeded by YAP1 was regained in miR-134-5p-transfected HTR8/SVneo cells (Figure 6C). Transfection of miR-134-5p alleviated the promotion effects of YAP1 on cells migration and invasion in HTR8/SVneo cells (Figure 6D and E). These data showed that overexpression of miR-134-5p counteracted the inhibition effects on cell migration and invasion, and accelerated the impact on cell apoptosis of HTR8/SVneo cells caused by YAP1.

### FAM99A positively regulated YAP1 expression by sponging miR-134-5p

**Figure 7:**
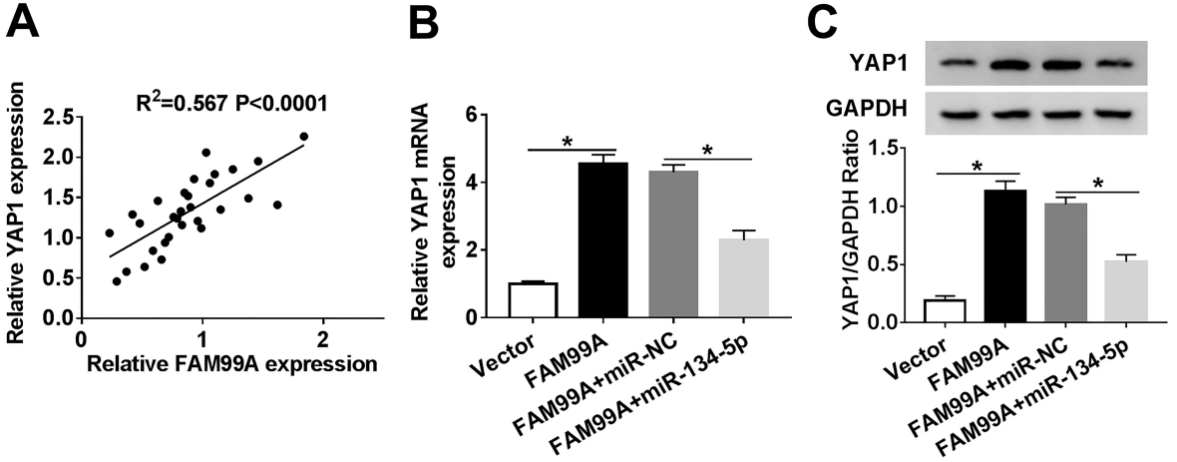
**A**, The correlation between YAP1 and FAM99A is shown. **B** and **C**, HTR8/SVneo cells were transfected with Vector, FAM99A, FAM99A+miR-NC (negative control), or FAM99A+miR-134-5p. **B**, The mRNA level of YAP1 was examined by qRT-PCR. **C**, The protein level of YAP1 was measured via western blot assay. Data are reported as means±SD. *P<0.05 (ANOVA).

YAP1 was positively correlated to FAM99A (Figure 7A). To investigate the mutual regulatory relationship of FAM99A, miR-134-5p, and YAP1 in PE, FAM99A and miR-134-5p were co-transfected into HTR8/SVneo cells. The mRNA and protein levels of YAP1 were enhanced in HTR8/SVneo cells transfected with FAM99A, but partially decreased with the re-introduction of miR-134-5p (Figure 7B and C). These data showed that FAM99A positively regulated YAP1 expression by targeting miR-134-5p.

## Discussion

PE is a threat for pregnant women worldwide. It is well known that lncRNAs play vital roles in diverse types of diseases. This research mainly focused on the mechanism of FAM99A in PE pathogenesis. These results demonstrated that FAM99A regulated cell behaviors of trophoblast cells in PE via the miR-134-5p/YAP1 axis.

Emerging evidence implies that dysregulation of lncRNAs is involved in PE pathogenesis. For example, lncRNA MALAT-1 depletion induces cell apoptosis *in vitro* (7). In PE, MEG3 (8), TUG1 (9), and PVT1 (10) exhibit similar results. In the present study, FAM99A was decreased in PE tissues and FAM99A overexpression boosted cell metastasis but impeded apoptosis in PE cells, which was in agreement with a previous report by He et al. (12). FAM99A is also associated with maternal circulating lipid profile in pregnant women (11). These data indicated that FAM99A plays a vital role in PE.

Accumulating evidence indicates that the aberrant expression of miR-134-5p is associated with many diseases. For instance, Wang et al. manifested that miR-134-5p was dramatically up-regulated in acute myocardial infarction (21). Another study reported that miR-134-5p impels podocyte cell apoptosis in diabetic nephropathy (22). In this study, miR-134-5p was validated as a direct target of FAM99A. miR-134-5p was enhanced in PE, and restrained cell metastasis but induced cell apoptosis *in vitro*, which were inhibited by FAM99A. Zou et al. (16) reported similar results in PE. These data indicated that FAM99A regulated cell behaviors by sponging miR-134-4p.

In previous research, YAP1 was linked to disease occurrence. For example, a study on congenital heart disease indicated that the low expression of YAP1 induces apoptosis (23). Ye et al. (24) demonstrated that YAP1 is lowly expressed in ventricular septal defects and its down-regulation retards cell growth. In the present study, YAP1 was verified to be a target of miR-134-5p. Moreover, YAP1 was remarkably reduced in PE. miR-134-5p decreased cell metastasis and promoted apoptosis by negatively regulating YAP1. Furthermore, FAM99A positively regulated YAP1 expression by sponging miR-134-5p. Our results are in agreement with those of Sun et al. (20) about the function of YAP1 in PE. These data revealed that FAM99A modulated YAP1 to affect behaviors of trophoblast cells in PE by sponging miR-134-5p.

In summary, the new FAM99A/miR-134-5p/YAP1 regulatory network was implicated in the pathogenesis of PE. Combined with the previous research, this complicated network may shed light on PE pathogenesis.

## References

[1] Steegers EA, Von Dadelszen P, Duvekot JJ, Pijnenborg R (2010). Pre-eclampsia. Lancet.

[B02] Ahmed A, Rezai H, Broadway-Stringer S (2017). Evidence-based revised view of the pathophysiology of preeclampsia. Adv Exp Med Biol.

[3] Hariharan N, Shoemaker A, Wagner S (2017). Pathophysiology of hypertension in preeclampsia. Microvasc Res.

[4] Chakraborty C, Gleeson LM, McKinnon T, Lala PK (2002). Regulation of human trophoblast migration and invasiveness. Can J Physiol Pharmacol.

[5] Tomimatsu T, Mimura K, Endo M, Kumasawa K, Kimura T (2017). Pathophysiology of preeclampsia: an angiogenic imbalance and long-lasting systemic vascular dysfunction. Hypertens Res.

[6] Schmitt AM, Chang HY (2016). Long noncoding RNAs in cancer pathways. Cancer Cell.

[7] Chen H, Meng T, Liu X, Sun M, Tong C, Liu J (2015). Long non-coding RNA MALAT-1 is downregulated in preeclampsia and regulates proliferation, apoptosis, migration and invasion of JEG-3 trophoblast cells. Int J Clin Exp Pathol.

[8] Zhang Y, Zou Y, Wang W, Zuo Q, Jiang Z, Sun M (2015). Down‐regulated long non‐coding RNA MEG3 and its effect on promoting apoptosis and suppressing migration of trophoblast cells. J Cell Biochem.

[9] Xu Y, Ge Z, Zhang E, Zuo Q, Huang S, Yang N (2017). The lncRNA TUG1 modulates proliferation in trophoblast cells via epigenetic suppression of RND3. Cell Death Dis.

[10] Xu Y, Lian Y, Zhang Y, Huang S, Zuo Q, Yang N (2018). The long non‐coding RNA PVT 1 represses ANGPTL 4 transcription through binding with EZH 2 in trophoblast cell. J Cell Mol Med.

[11] Petry CJ, Koulman A, Lu L, Jenkins B, Furse S, Prentice P (2018). Associations between the maternal circulating lipid profile in pregnancy and fetal imprinted gene alleles: a cohort study. Reprod Biol Endocrinol.

[12] He T, Qiao Y, Lv Y, Wang J, Hu R, Cao Y (2019). lncRNA FAM99A is downregulated in preeclampsia and exerts a regulatory effect on trophoblast cell invasion, migration and apoptosis. Mol Med Rep.

[13] Mohr AM, Mott JL (2015). Overview of microRNA biology. Semin Liver Dis.

[14] Zhang Y, Fei M, Xue G, Zhou Q, Jia Y, Li L (2012). Elevated levels of hypoxia‐inducible microRNA‐210 in pre‐eclampsia: new insights into molecular mechanisms for the disease. J Cell Mol Med.

[15] Wang W, Feng L, Zhang H, Hachy S, Satohisa S, Laurent LC (2012). Preeclampsia up-regulates angiogenesis-associated microRNA (i.e., miR-17,-20a, and-20b) that target ephrin-B2 and EPHB4 in human placenta. J Clin Endocrinol Metab.

[16] Zou AX, Chen B, Li QX, Liang YC (2018). MiR-134 inhibits infiltration of trophoblast cells in placenta of patients with preeclampsia by decreasing ITGB1 expression. Eur Rev Med Pharmacol Sci.

[17] Kodaka M, Hata Y (2015). The mammalian Hippo pathway: regulation and function of YAP1 and TAZ. Cell Mol Life Sci.

[18] Zhao X, Sun J, Chen Y, Su W, Shan H, Li Y (2018). lncRNA PFAR promotes lung fibroblast activation and fibrosis by targeting miR-138 to regulate the YAP1-twist axis. Mol Ther.

[19] Ou C, Sun Z, He X, Li X, Fan S, Zheng X (2019). Targeting YAP1/LINC00152/FSCN1 signaling axis prevents the progression of colorectal cancer. Adv Sci (Weinh).

[20] Sun M, Na Q, Huang L, Song G, Jin F, Li Y (2018). YAP is decreased in preeclampsia and regulates invasion and apoptosis of HTR-8/SVneo. Reprod Sci.

[21] Wang KJ, Zhao X, Liu YZ, Zeng QT, Mao XB, Li SN (2016). Circulating MiR-19b-3p, MiR-134-5p and MiR-186-5p are promising novel biomarkers for early diagnosis of acute myocardial infarction. Cell Physiol Biochem.

[22] Qian X, Tan J, Liu L, Chen S, You N, Yong H (2018). MicroRNA-134-5p promotes high glucose-induced podocyte apoptosis by targeting bcl-2. Am J Transl Res.

[23] Zheng J, Peng B, Zhang Y, Ai F, Hu X (2019). miR-9 knockdown inhibits hypoxia-induced cardiomyocyte apoptosis by targeting Yap1. Life Sci.

[24] Ye L, Yin M, Xia Y, Jiang C, Hong H, Liu J (2015). Decreased yes-associated protein-1 (yap1) expression in pediatric hearts with ventricular septal defects. PloS One.

